# Flavonol glycosides accumulation in faba bean grown under combined selenium and sulfur application

**DOI:** 10.1007/s11306-025-02313-4

**Published:** 2025-08-12

**Authors:** Muna Ali Abdalla, Khuram Waqas, Susanne Neugart, Karl Hermann Mühling

**Affiliations:** 1 Institute of Plant Nutrition and Soil Science, Kiel University, Hermann- Rodewald-Str. 2, 24118 Kiel, Germany; 2 Department of Crop Sciences, Division of Quality and Sensory of Plant Products, Georg-August-Universität Göttingen, Carl-Sprengel-Weg 1, 37075 Göttingen, Germany

**Keywords:** Legume–*Rhizobium*, symbiosis, Selenium, Sulfur, Chalcone synthase, Flavonol glycosides

## Abstract

**Introduction:**

Faba bean (*Vicia faba* L.) leaves are edible; hence, they are primarily used as animal feed in agriculture. Additionally, seed pods and other plant tissues are considered edible and are used as green vegetables in many parts of the world.

**Objectives:**

Flavonol glycosides are well-known in faba bean leaves; accordingly, in this study, we followed a targeted metabolomic approach to explore glycosylated flavonols and their concentrations in response to contrasting levels of selenium (Se) and sulfur (S) enrichment under faba bean–*Rhizobium *symbiosis.

**Methods:**

Faba bean plants were cultivated under growth chamber conditions and enriched with different levels of selenium and sulfur under *Rhizobium* inoculation. Their leaves were extracted using 70% methanol to quantify glycosylated flavonoids. Sample leaves were analyzed through a targeted method using high-performance liquid chromatography combined with a diode array detector (HPLC-DAD) and electrospray ionization-quadrupole-time-of-flight tandem mass spectrometry detection (HPLC-ESI-Q-ToF-MS/MS).

**Results:**

The analysis led to semi-quantifying 11 flavonol glycosides. Analysis of the metabolites of the different faba bean leaf extracts confirmed that selenium has a considerable impact on the accumulation of flavonol glycosides, especially under sulfur availability, possibly because it induces chalcone synthase and other enzymes for flavonols’ biosynthesis.

**Conclusion:**

To the best of our knowledge, this is the first report to investigate the impact of selenium and sulfur enrichment on the accumulation of faba bean flavonols under atmospheric nitrogen (N_2_) fixation conditions. This study highlights the medicinal and nutritional benefits of legumes as an essential source of protein in plant-based foods.

## Introduction

In recent years, global agricultural efficiency and productivity have contributed to increased food production, enabling a rapidly growing population worldwide to be fed. Accordingly, various chemical nitrogen (N) fertilizers—known as one of the greatest inventions of the twentieth century to keep almost half the world’s population alive—are synthesized from ammonia (Erisman et al. 2008). However, the application of synthetic N fertilizers to cropland has become a source of numerous environmental problems; hence, a high proportion of N loss from agricultural systems to the environment substantially affects biodiversity and terrestrial and aquatic ecosystems (Sutton et al. 2011). Symbiotic N fixation is a process in which atmospheric N is converted into ammonia by diazotrophic prokaryotes (e.g. rhizobia) and constitutes one of the strategies to improve agriculture’s sustainability by enhancing agricultural production with low environmental and economic costs (Xu and Wang 2023). Only legumes are known to form symbioses with rhizobia, as an integral source of fixed nitrogen in agricultural lands (Mathesius 2022).

Faba bean (*Vicia faba *L. Leguminosae) is concurrently one of the foremost legume crops that enhance sustainability and a global source of plant protein used for food and feed. Additionally, it contains dietary fiber, complex carbohydrates, and essential vitamins and minerals for human health, including folate, iron, zinc, phosphorus, and magnesium (Martineau-Côté et al. 2022; Méndez-López et al. 2022). Moreover, faba bean is the staple crop for approximately 400 million inhabitants in North and East Africa, mainly in Ethiopia, Egypt, Sudan, Morocco, and Tunisia (Maalouf et al. 2021. Additionally, faba bean plants are edible and known as novel green vegetables in many other countries (Etemadi et al. 2019; Renna et al. 2018). Moreover, seed pods and other plant parts have medicinal significance as a Parkinson’s Disease therapy due to their l-DOPA content (Silva et al. 2024.

A recent study demonstrated that the leaves and flowers of faba beans, showed higher concentrations of levodopa, a non-protein amino acid, widely used in the treatment of Parkinson’s disease (LeWitt and Fahn 2016). Moreover, these parts exhibited low amounts of vicine, an antinutritional substance, related to the pyrimidine glycosides class. Accordingly, the authors suggested that fresh plant tissues are an alternative, plant-based source of l-DOPA, playing a remarkable role as a food-based treatment for Parkinson’s Disease (Silva et al. 2024).

Sulfur is a crucial macronutrient that supports physiological processes in plants. Supplementing food crops with sulfur directly affects their nutritional quality by influencing the synthesis of primary (e.g. amino acids, sulfolipids, vitamins, proteins) and secondary metabolites (e.g. glucosinolates and phytoalexins). Selenium is a trace element that is essential for human and animal health. Since the discovery of glutathione peroxidase—which constitutes one of the critical selenoproteins in the human body that directly helps control free radicals and reduce the inflammatory response—selenium is involved in many physiological processes. Furthermore, it benefits plant growth and development by improving a plant’s resistance to various abiotic stresses. The physicochemical similarities between selenium and sulfur, as well as their similarities in the pathways through which they are absorbed, translocated, and assimilated, attract research interest in understanding the impact of their interaction on the overall quality profile of food crops (Abdalla and Mühling 2023).

**Fig. 1:**
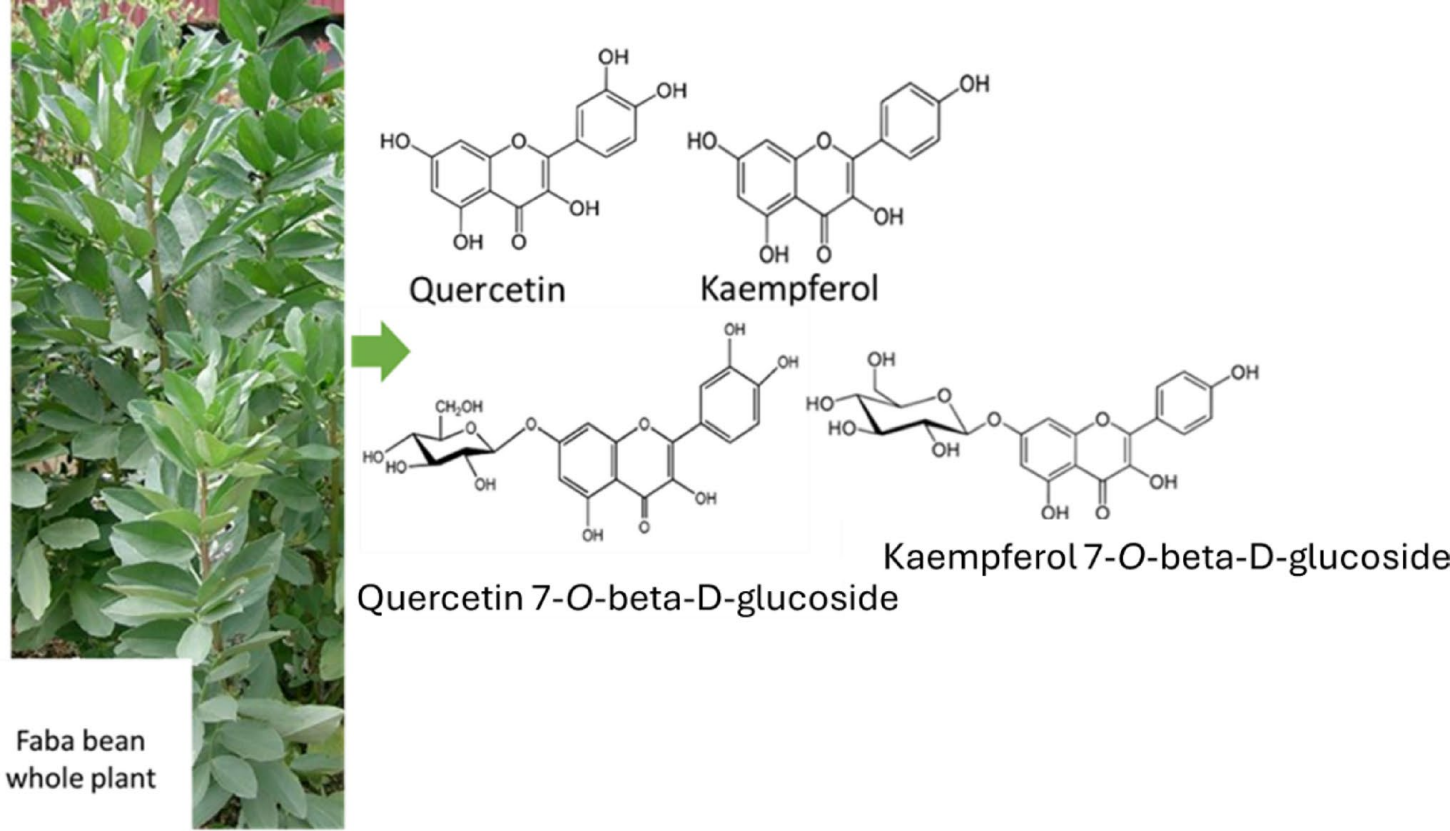
Quercetin, kaempferol, and their monoglycosides isolated from faba bean (Tomás-Lorente et al. 1990)

Flavonoids constitute a large group of biologically active secondary metabolites derived from the phenylpropanoid pathway. They consist of two aromatic rings (A and B) connected with a heterocyclic ring (C) bearing an oxygen atom. They are mainly conjugated with one or more sugar molecules and are called flavonoid glycosides. The use of different classes of flavonoid glycosides is widespread in leguminous plants; for instance, flavone glycosides have been found in the leaves of *Lupinus angustifolius *(Duenas et al. 2009) and *Medicago truncatula *(Alvarez-Rivera et al. 2022). In addition, isoflavones are mostly present in the seeds of *Glycine max*L. (Sohn et al. 2021), *Lens culinaris*, *Phaseolus vulgaris*, *Cicer arietinum *(Konar et al. 2012) *M. truncatula* (Alvarez-Rivera et al. 2022). Moreover, faba bean leaves are a well-recognized source of flavonols (Neugart et al. 2015). For instance, faba bean leaves have significant amounts of mono- (Fig. 1), di-, tri-, and tetraglycosides of kaempferol and quercetin—which are often associated with sugar moieties, such as glucose, rhamnose, and galactose—and those of nonacylated and acetylated kaempferol glycosides (Neugart et al. 2015; Spanou et al. 2012).

As important ingredients in human diets, flavonoids have demonstrated impressive therapeutic properties that diminish the burden of various diseases (e.g. antidiabetic, antimicrobial, anti-inflammatory, and anticancer bioactivities) (Dias et al. 2021;Dixon and Pasinetti 2010; Ullah et al. 2020; Shen et al. 2022). Flavonoids possess remarkable antioxidant capacity and can reduce or inhibit oxidative stress caused by free radicals through multiple mechanisms, including direct scavenging of reactive oxygen species (ROS), activation of antioxidant enzymes, and inhibition of oxidative enzymes that generate ROS (Williamson et al. 2018; Xiang et al. 2018; Shen et al. 2022). Therefore, flavonoids play an essential role in plant responses to various stresses and in human health.

Advancements in Liquid Chromatography-Mass Spectrometry (LC-MS) techniques enable the improved identification and quantification of secondary metabolites (e.g. flavonoids, alkaloids, terpenoids, etc.) through the application of high-resolution mass spectrometry (HRMS). HPLC-DAD-ESI-QToF-MS is a powerful technique in targeted and untargeted metabolomics (Bai and Li 2024). Furthermore, separation with high resolution using Q-ToF-MS/MS scanning modes enables the detection of both fragment ions and intact molecular ions, allowing for a higher spectral acquisition rate and the structural determination of bioactive compounds (Holčapek et al. 2012).

Sulfur is important for N_2_fixation as a component of iron–sulfur (Fe–S) clusters of nitrogenase—an enzyme that converts atmospheric N into ammonia (Becana et al. 2018; Kalloniati et al. 2015; Varin et al. 2010). Moreover, sulfur is important for the accumulation of cytoplasmic leghemoglobin, which facilitates the oxygen supply to the bacteria and prevents nitrogenase from being inactivated under high oxygen conditions (Singh and Varma, 2017; Becana et al. 2018). In a previous study, less leghemoglobin was found under sulfur deprivation, which inhibited rhizobia nitrogenase (Varin et al. 2010).

Research on the effective role of selenium in maximizing legume–*Rhizobium* symbiosis—a potential approach to achieving sustainable agriculture—has been limited (Abdalla et al. 2025). In this study, we have drawn research attention to the metabolic changes associated with selenium application in the presence of adequate or higher sulfur levels; however, crosstalk between these two elements can impact plant biochemical processes. Therefore, in this study, we hypothesized that the enrichment of selenium in the presence of adequate sulfur increases the accumulation of flavonol glycosides under faba bean–*Rhizobium* symbiosis. Hence, selenium and sulfur have an antagonistic interaction; sufficient sulfur is important for efficient faba bean–*Rhizobium* symbiosis, required for the better growth and productivity of faba bean. Accordingly, the underlying mechanism in this regard could be that under sulfur availability, selenium upregulates genes within the phenylpropanoid pathway (e.g. chalcone synthase (CHS) and chalcone isomerase (CHI), to biosynthesize naringenin, the precursor for flavonol glycosides and other *C*-glycosylated flavones.

## Materials and methods

### Treatment and experimental design

The experiment was conducted at the Institute of Plant Nutrition and Soil Science, Kiel University, Kiel, Germany. The faba bean cultivar Mallory was hydroponically grown in a controlled climatic chamber under the following conditions: temperature 20/15°C, relative humidity 50/60%, and light 200 µmol m^−2^ s^−1^ PPFD with 14/10 h duration for day/night, respectively. The seeds were surface-sterilized with 2% sodium hypochlorite for 5 min. Then, they were spread into sandwich blots, rinsed with deionized water, and placed in a container containing 0.5 mM CaCl_2_ to facilitate germination. Subsequently, the growing seedlings were transferred into the 5 L pots after 10 days. The basal nutrient solution was applied equally and changed twice a week. The basal nutrient solution consisted of macronutrients (mM), including CaCl_2_ = 1; MgCl_2_ = 0.5; K_2_HPO_4_ = 0.5; KH_2_PO_4_ = 0.5, and micronutrients (µM), including Fe-EDTA = 200; H_3_BO_3_ = 10; MnSO_4_ = 2; ZnSO_4_ = 0.5; CuSO_4_ = 0.3; (NH_4_)_2_Mo_7_O_24_ = 0.01, and NiSO_4_ = 0.005. In addition, NH_4_NO_3_ (1 mM) was added once as a starter to facilitate the seedlings’ growth until nodulation. Each plant was inoculated with 1 mL *Rhizobium leguminosarum* bv. *viciae *3841 inoculum that was prepared as suggested by (Pastor-Bueis et al. 2019). To investigate the impact of selenium and sulfur enrichment on flavonoid accumulation during faba bean–*Rhizobium* symbiosis, three different levels of sulfur (S0; 0 mM, S1: 0.5 mM, and S2; 1.5 mM (K_2_SO_4_) were applied with three different selenium levels (Se0; 0 µM, Se; 2 µM and Se2; 5 µM (Na_2_SeO_4_). Sulfur treatments were applied at the time of transplantation, while selenium levels were added 21 days post-transplantation. Subsequently, nine treatments were performed, and each treatment had four replicates arranged in a completely randomized design. Plants were harvested after 8 weeks of transplantation. Shoots of the plants were washed thoroughly with deionized water and immediately frozen in liquid nitrogen and freeze-dried (Gamma1–20, Christ, Osterode am Harz, Germany). The dried leaves were ground into a fine powder and kept for the analysis of the metabolites.

### Sample extraction

The powdered leaves were extracted with 70% methanol (500 mL), and the organic solvent was removed using a rotary evaporator. Finally, the resulting crude extracts were stored at − 20 °C before metabolite analysis.

### HPLC-DAD-ESI-Q-ToF MS/MS analysis

**Fig. 2:**
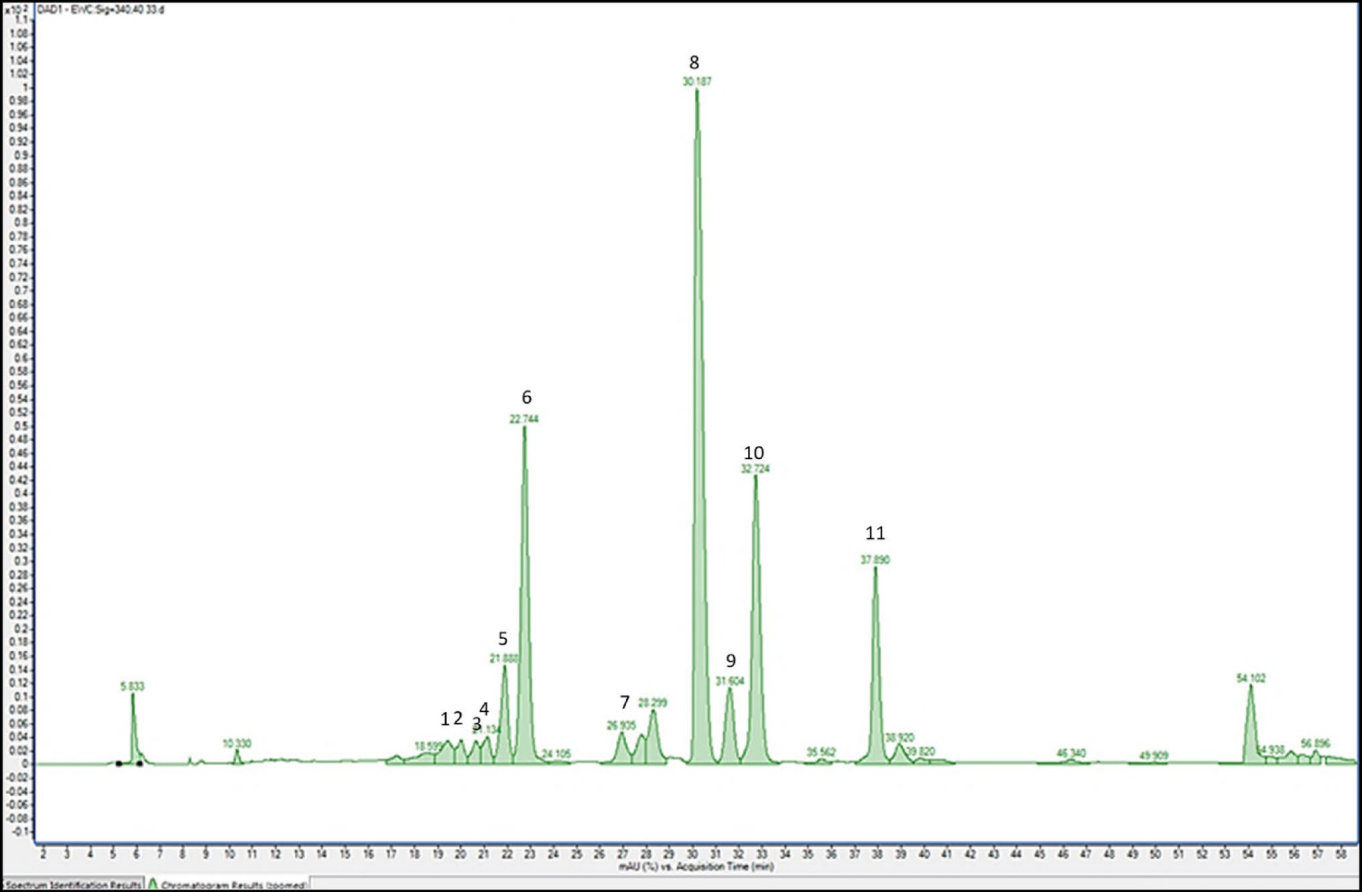
HPLC-DAD chromatogram of the flavonol glycosides in faba bean plants enriched with contrasting levels of selenium and sulfur under nitrogen fixation conditions

The crude extract (redissolved in 60% acidified methanol) was used to analyze the flavonoid compounds. Flavonoid composition (including hydroxycinnamic acid derivatives and flavonoid glycosides) and concentrations were determined from a filtrate using a series 1290 HPLC (Agilent Technologies, Waldbronn, Germany) equipped with a degasser’s vacuum chamber, binary pump, autosampler, column oven, and photodiode array detector. The compounds were separated at 25 °C using a Sphinx RP column (150 mm × 4.6 mm, 5 μm, NUCLEODUR^®^). Eluent A was 0.5% acetic acid, and eluent B was 100% acetonitrile. The gradient for eluent B was adjusted as follows: 5–12% (0–3 min), 12–25% (3–46 min), 25–90% (46–49.5 min), 90% isocratic (49.5–52 min), 90–5% (52–52.7 min), and 5% isocratic (52.7–59 min). Metabolites were determined at a flow rate of 0.3 mL min^−1^and wavelengths of 280 nm, 320 nm, 330 nm, 370 nm, and 520 nm. The glycosides of flavonoids derivatives were tentatively identified as deprotonated molecular ions and characteristic mass fragment ions according to (Neugart et al. 2015; Schmidt et al. 2010) by HPLC-DAD-ESI-Q-ToF using an Agilent 6545 Q-Tof in negative ionization mode. For the identification of the peaks (Fig.2), the data were compared to those of the literature related to the investigated species and their relatives. In the mass spectrometer, nitrogen was used as the dry gas (8 L min^−1^, 325 °C) and nebulizer gas (40 psi), with a capillary voltage of − 4000 V. Standards (Quercertin 3-glucoside, and kaempferol 3-glucoside, Roth, Karlsruhe, Germany) were used for external calibration curves using a semi-quantitative approach. The results are provided as mg g^−1^ dry weight. Each sample was analyzed in four separate replicates.

### Statistical analysis

A two-way ANOVA was used to evaluate the variations in flavonol glycoside concentrations according to the levels of the two independent variables (selenium x sulfur). Tukey’s honestly significant difference (HSD) test was performed at a significance level of 5%. Calculations were performed using Statistics 10.

## Results and discussion

### Structural identification and quantification of flavonol glycosides

Eleven kaempferol and quercetin glycosylated derivatives were detected in all treatments and were tentatively identified; among them, two were further acylated flavonol glycosides. The identified flavonol glycosides are: kaempferol 3-*O*-rhamnogalactoside 7-*O*-rhamnoside (peak 1), quercetin 3-*O*-rhamnoglucoside 7-*O*-rhamnoside (peak 2), kaempferol 3-*O*-rhamnoglucoside 7-*O*-rhamnoside 4′-*O*-rhamnoside (isomer 1) (peak 3), kaempferol 3-*O*-rhamnoglucoside 7-*O*-rhamnoside 4′-*O*-rhamnoside (isomer 2) (peak 4), kaempferol 3-*O*-rhamnoglucoside 7-*O*-rhamnoside (isomer 1) (peak 5), kaempferol 3-*O*-rhamnoglucoside 7-*O*-rhamnoside (isomer 2) (peak 6), quercetin 3-*O*-rhamnoarabinoside 7-*O*-rhamnoside (peak 7), kaempferol 3-*O*-rhamnoarabinoside 7-*O*-rhamnoside (peak 8), kaempferol 3-*O*-acetyl-rhamnogalactoside 7-*O*-rhamnoside (isomer 1) (peak 9), kaempferol 3-*O*-acetyl-rhamnogalactoside 7-*O*-rhamnoside (isomer 2) (peak 10), and kaempferol 3-*O*-rhamnoglucoside (peak 11) as shown in Fig. 2; Table 1. Among all detected flavonol glycosides, kaempferol 3-*O*-rhamnoarabinoside 7-*O*-rhamnoside was the most abundant compound, with differences among all treatments (Fig. 2, peak 8). Kaempferol 3-*O*-rhamnoglucoside 7-*O*-rhamnoside (isomer 2) (Fig. 2, peak 6) and kaempferol 3-*O*-acetyl-rhamnogalactoside 7-*O*-rhamnoside (isomer 2) (Fig. 2, peak 10) were the second most abundant compounds. (Fig. 2). The mass spectra of all detected compounds delivered the deprotonated molecular ions [M–H]^−^ (Tables 1 and 2). Table 1 displays the peaks according to their mass spectra, maximum absorbance wavelength (λ_max_ (nm) in their UV–VIS spectra, and retention times (RT). Table 2 shows spectral data, including monoisotopic masses, theoretical and observed *m/z* values, and characteristic product ions after CID, which characterized the flavonal glycosides through detailed comparison with literature data (Neugart et al. 2015; Spanou et al. 2008, 2012; Tomas-Lorente et al. 1989).

Two acylated kaempferol glycosides were identified in the leaves of faba bean in the current study. The peak observed at RT = 31.55 min, which was tentatively characterized, presented a deprotonated *pseudo* molecular ion [M − H]^−^ at *m/z* 781.21986 (mass error = −0.95 ppm). The detected characteristic fragments after CID were *m/z* 635.16547 [M-H-rha]^−^ (mass error = 6.71 ppm), 430.09657 [M-2H-rha-gal-AcOH]^−^ (mass error = 4.77 ppm) and 284.03425 [M-2H-2rha-gal-AcOH]^−^ (mass error = 5.02 ppm). Based on previously reported NMR spectroscopic analysis of flavonol glycosides from the leaves of faba bean (Spanou et al. 2008, 2012; Tomas-Lorente et al. 1989), this compound was identified as kaempferol-3-*O*-acetyl-galactoside-7-*O*-rhamnoside. Furthermore, the second peak at RT = 32.68 min corresponds to an isomer of this compound, characterized by comparable deprotonated *pseudo* molecular ions and characteristic fragments. The mass accuracy and MS/MS data are listed in Table 2.

Regarding the assignment of the glycosylated position, all mass spectra were commonly characterized by the presence of deprotonated molecular ions [M − H]^−^. The structure elucidation of sugar moieties and hydroxycinnamic acid substitutions at the flavonoid aglycone was performed by comparing the MS spectral data with literature, mainly conducted on flavonoids glycosides in kale and faba beans (Neugart et al. 2015; Schmidt et al. 2010). The published reports demonstrated that the initial fragmentation of the [M − H]^−^ ion typically involves cleavage of the *O*-glycosidic bond at the 7-*O* position, while any remaining sugars are attached to the hydroxyl group at the 3-*O* position of the flavonol backbone (Schmidt et al. 2010; Vallejo et al. 2004). Flavonoid diglycosides with sugars linked to different hydroxyl positions yield characteristic fragment ions: [M − H−162]^−^ for glucose or galactose, [M − H−146]^−^ for rhamnose, and [M  H−132]^−^ for arabinose, with these ions often appearing as base peaks (Ferreres et al. 2004). Additionally, flavonoid glycosides acylated with acetic acid can be identified by a neutral loss of 60 Da (Neugart et al. 2015).

**Table 1:** Molecular weight, molecular formula, λ_max_ (nm), and MS fragmentation patterns of flavonol glycosides in faba beans

Compound	MW	Molecular formula	Peak no	RT	λ_max_ (nm)	MS	MS^2^
						[M–H]^−^	
Kaempferol 3-*O*-rhamnogalactoside 7-*O*-rhamnoside	740	C_33_H_40_O_19_	1	19.416	264,344	739	593,430,284
Quercetin 3-*O*-rhamnoglucoside 7-*O*-rhamnoside	756	C_33_H_40_O_20_	2	19.949	256,332	755	446,609,299
Kaempferol 3-*O*-rhamnoglucoside 7-*O*-rhamnoside 4′-*O-*rhamnoside (isomer 1)	886	C_39_H_50_O_23_	3	20.604	264,340	885	739,284,430
Kaempferol 3-*O*-rhamnoglucoside 7-*O*-rhamnoside 4′-*O-*rhamnoside (isomer 2)	886	C_39_H_50_O_23_	4	21.108	264,340	885	739,284,430
Kaempferol 3-*O*-rhamnoglucoside 7-*O*-rhamnoside (isomer 1)	740	C_33_H_40_O_19_	5	21.866	252,264,344	739	593,430,284
Kaempferol 3-*O*-rhamnoglucoside 7-*O*-rhamnoside (isomer 2)	740	C_33_H_40_O_19_	6	22.725	252,264,344	739	593,430,284
Quercetin 3-*O*-rhamnoarabinoside 7-*O*-rhamnoside	726	C_32_H_38_O_16_	7	26.873	268,340	725	446,579,299
Kaempferol 3-*O*-rhamnoarabinoside 7-*O*-rhamnoside	710	C_32_H_38_O_18_	8	30.181	268,352	709	563,430,284
Kaempferol 3-*O*-acetyl-rhamnogalactoside 7-*O*-rhamnoside isomer 1	782	C_35_H_42_O_20_	9	31.556	236,268,336	781	635,430,284
Kaempferol 3-*O*-acetyl-rhamnogalactoside 7-*O*-rhamnoside (isomer 2)	782	C_35_H_42_O_20_	10	32.686	236,268,336	781	635,430,284
Kaempferol 3-*O*-rhamnoglucoside	594	C_27_H_30_O_15_	11	38.861	268,328	593	284,429

**Table 2:** Monoisotopic masses, theoretical and observed *m/z* values, and characteristic product ions after CID of flavonol glycosides in faba beans

	Compound name										
	K 3-*O*-rhagal 7-*O*-rha (peak 1)	Q 3-*O*-rhaglc 7-*O*-rha (peak 2)	K 3-*O*-rhaglc 7-*O*-rha-4′-*O*-rha (isomer 1) (peak 3)	K 3-*O*-rhaglc 7-*O*-rha-4′-*O*-rha (isomer 2) (peak 4)	K 3-*O*-rhaglc 7-*O*-rha (isomer 1) (peak 5)	K 3-*O*-rhaglc 7-*O*-rha (isomer 2) (peak 6)	Q 3-*O*-rhaara 7-*O*-rha (peak 7)	K 3-*O*-rhaara 7-*O*-rha (peak 8)	K 3-*O*-acetyl -rhagal 7-*O*-rha (isomer 1) (peak 9)	K 3-*O*-acetyl -rhagal 7-*O*-rha (isomer 2) (peak 10)	K 3-*O*-rhaglc (peak 11)
Monoisotopic mass	740.21638	756.21129	886.27429	886.27429	740.21638	740.21638	726.20073	710.20581	782.22694	782.22694	594.15847
Theoretical *m/z* [M-H] ^−^	739.20855	755.20347	885.26646	885.26646	739.20855	739.20855	725.19290	709.19799	781.21912	781.21912	593.15065
Observed *m/z*	739.20972	755.20424	885.26699	885.26681	739.20902	739.20984	725.19578	709.19946	781.21986	781.22015	593.15104
Mass Error (ppm)	1.58	1.02	0.60	0.40	0.64	1.74	3.97	2.07	0.95	1.32	0.66
Characteristic product ions after collision-induced dissociation (CID)	593.15348 [M-H-rha]^−^ (mass error = 4.77 ppm) 430.09254 [M-2H-rha-gal]^−^ (mass error = 5.91 ppm) 284.03488 [M-2H-2rha-gal]^−^ (mass error = 6.49 ppm)	609.14878 [M-H-rha]^−^ (mass error = 5.28 ppm) 446.08741 [M-2H-rha-gal]^−^ (mass error = 5.81 ppm) 299.02141[M-2H-2rha-gal]^−^ (mass error = 5.18 ppm)	739.21198 [M-H-rha]^−^ (mass error = 4.64 ppm) 430.09195 [M-2H-2rha-glc]^−^ (mass error = 4.53 ppm) 284.03402 [M-2H-3rha-glc]^−^ (mass error = 4.49 ppm)	739.21115 [M-H-rha]^−^ (mass error = 3.52 ppm) 430.09214 [M-2H-2rha-glc]^−^ (mass error = 4.98 ppm) 284.03397 [M-2H-3rha-glc]^−^ (mass error = 4.37 ppm)	593.15298 [M-H-rha]^−^ (mass error = 3.93 ppm) 430.09145 [M-2H-rha-glc]^−^ (mass error = 3.37 ppm) 284.03427 [M-2H-2rha-glc]^−^ (mass error = 5.07 ppm)	593.15304 [M-H-rha]^−^ (mass error = 4.03 ppm) 430.09241 [M-2H-rha-glc]^−^ (mass error = 5.60 ppm) 284.03471 [M-2H-2rha-glc]^−^ (mass error = 6.09 ppm)	579.13787 [M-H-rha]^−^ (mass error = 4.96 ppm) 446.08807 [M-2H-rha-ara]^−^ (mass error = 7.35 ppm) 299.02185 [M-2H-2rha-ara]^−^ (mass error = 6.21 ppm)	563.13742 [M-H-rha]^−^ (mass error = 4.72 ppm) 430.09287 [M-2H-rha-ara]^−^ (mass error = 6.67 ppm) 284.03399 [M-2H-2rha-ara]^−^ (mass error = 4.42 ppm)	635.16547 [M-H-rha]^−^ (mass error = 6.71 ppm) 430.09657 [M-2H-rha-gal-AcOH]^−^ (mass error = 4.77 ppm) 284.03425 [M-2H-2rha-gal-AcOH]^−^ (mass error = 5.02 ppm)	635.16426 [M-H-rha]^−^ (mass error = 4.80 ppm) 430.09185 [M-2H-rha-gal-AcOH]^−^ (mass error = 4.30 ppm) 284.03388 [M-2H-2rha-gal-AcOH]^−^ (mass error = 4.16 ppm)	430.09257 [M-2H-glc]^−^ (mass error = 5.98 ppm) 284.03452 [M-2H-rha-glc]^−^ (mass error = 5.65 ppm)

*Theoretical *m/z* of the characteristic product ions after collision-induced dissociation (CID)

[M-H]^−^ K + Rha + Ara 563.14008; Q + Rha + Ara 579.13500; K + Rut 593.15065; Q + Rut 609.14556; K + Rha + Gal + AcOH 635.16121; K + Rut + Rha 739.20855

[M-2H]^−^ Kaempferol (K) 284.03209; Quercetin (Q) 300.02700; K + Rha 430.09000; Q + Rha 446.08491

[M-3H]^−^ Quercetin (Q) 299.01918

The accumulation of the various metabolites is depicted in Table 3. Quercetin 3-*O*-rhamnoglucoside 7-*O*-rhamnoside and quercetin 3-*O*-rhamnoarabinoside 7-*O*-rhamnoside were the main quercetin glycosides in all samples. Kaempferol glycosides were found as the most abundant flavonols, with only two quercetin glycosides present in minor levels. Hence, the cultivar used in this study “Mallory” showed better performance in winter and spring, the current findings agreed with Yan et al. (2018), who reported that kaempferol glycosides were the major flavonoids compared to quercetin in the leaves of German winter (Hiverna and Nordica) and spring (Fuego and Espresso) cultivars cultivated under greenhouse conditions.

Quercetin 3-*O*-rhamnoglucoside 7-*O*-rhamnoside concentration increased by 13.3% and 18.8% in response to lower selenium (Se1 = 2 µM) and adequate sulfur (S1 = 0.5 mM) levels and higher selenium (Se2 = 5 µM) and sulfur (S2 = 0.5 mM) treatments, respectively. However, the quercetin 3-*O*-rhamnoarabinoside 7-*O*-rhamnoside concentration remained unaffected under various selenium and sulfur treatments.

In a previous report, a high concentration of kaempferol 3-*O*-rhamnoarabinoside 7-*O*-rhamnoside was found in the leaves of summer cultivars of faba beans for the first time (Neugart et al. 2015). This finding was consistent with the current report, which identified kaempferol 3-*O*-rhamnoarabinoside 7-*O*-rhamnoside as the most abundant compound among all detected metabolites. The concentration of kaempferol 3-*O*-rhamnoarabinoside 7-*O*-rhamnoside adequate or higher sulfur levels with lower or higher selenium levels increased drastically by 53% and 51%, respectively. Among the kaempferol glycosides, the kaempferol 3-*O*-rhamnogalactoside 7-*O*-rhamnoside concentration remained unaffected by varied selenium and sulfur levels. Kaempferol 3-*O*-rhamnoglucoside 7-*O*-rhamnoside 4′-*O*-rhamnoside (isomer 1) concentrations were elevated by 44% and 54% in the availability of adequate and higher sulfur levels, respectively, with no selenium treatment (Se0S1) and (Se0S2). Isomer 2 of the same compound increased considerably by 48% and 60% in response to lower and higher selenium levels with adequate and higher sulfur application, respectively. In addition, kaempferol 3-*O*-rhamnoglucoside 7-*O*-rhamnoside (isomer 1) exhibited a 50% accumulation with higher sulfur levels and no selenium treatment. Its concentration also increased by 46%, and 48% with the same sulfur level and lower and higher selenium levels, respectively. Moreover, isomer 2 of the same compound increased by 38% and 52% with lower selenium and adequate sulfur (Se1S1) and higher selenium and sulfur (Se2S2) levels, respectively. Kaempferol 3-*O*-acetyl-rhamnogalactoside 7-*O*-rhamnoside (isomer 1) increased by 31% and 49% with a sufficient and higher sulfur supply with no selenium treatment, respectively. In addition, lower or higher selenium levels under both sulfur levels did not significantly affect this compound. The accumulation of isomer 2 of the same compound increased by 47% with higher sulfur and lower selenium treatments. Moreover, the kaempferol 3-*O*-rhamnoglucoside concentration increased by 50% with adequate sulfur and no selenium treatments. Moreover, it enhanced by (57%) under lower selenium and sufficient sulfur application.

**Table 3:** The concentration of flavonol glycosides (mg g^−1^ DM) of faba bean leaves grown with different selenium (Se0; 0 µM, Se1; 2 µM and Se2; 5 µM, Na_2_SeO_4_) and sulfur (S0; 0 mM, S1: 0.5 mM, and S2; 1.5 mM, K_2_SO_4_) levels in combination with *Rhizobium*

Compound no.	S0			S1			S2		
	Se0	Se1	Se2	Se0	Se1	Se2	Se0	Se1	Se2
Kaempferol 3-*O*-rhamnogalactoside 7-*O*-rhamnoside	0.11 ± 0.03 AB	0.05 ± 0.01 **C**	0.09 ± 0.02 BC	0.11 ± 0.01 AB	0.12 ± 0.01 AB	0.13 ± 0.02 AB	0.15 ± 0.04 A	0.14 ± 0.01 AB	0.15 ± 0.01 A
Quercetin 3-*O*-rhamnoglucoside 7-*O*-rhamnoside	0.0013 ± 0.01 CD	0.0013 ± 0.01 BCD	0.0013 ± 0.01 D	0.0013 ± 0.01D	0.0015 ± 0.01AB	0.0015 ± 0.02ABC	0.0015 ± 0.01AB	0.0016 ± 0.01 A	0.0016 ± 0.01 A
Kaempferol 3-*O*-rhamnoglucoside 7-*O*-rhamnoside 4′-*O*-rhamnoside (isomer 1)	0.05 ± 0.01 C	0.07 ± 0.0 BC	0.05 ± 0.01 C	0.09 ± 0.01AB	0.08 ± 0.01ABC	0.07 ± 0.01ABC	0.11 ± 0.01 A	0.10 ± 0.01AB	0.11 ± 0.0 A
Kaempferol 3-*O*-rhamnoglucoside 7-*O*-rhamnoside 4′-*O*-rhamnoside (isomer 2)	0.044 ± 0.01 F	0.056 ± 0.00 EF	0.07 ± 0.00 CDE	0.065 ± 0.00 DEF	0.085 ± 0.02 BCD	0.077 ± 0.01 BCDE	0.01 ± 0.02 AB	0.11 ± 0.00 A	0.09 ± 0.00 ABC
Kaempferol 3-*O*-rhamnoglucoside 7-*O*-rhamnoside (isomer 1)	0.63 ± 0.17 B	0.75 ± 0.1 B	0.82 ± 0.11 B	0.82 ± 0.01 B	0.84 ± 0.13 B	0.82 ± 0.05 B	1.26 ± 0.08 A	1.17 ± 0.16 A	1.22 ± 0.11 A
Kaempferol 3-*O*-rhamnoglucoside 7-*O*-rhamnoside (isomer 2)	0.18 ± 0.06 C	0.22 ± 0.01 BC	0.22 ± 0.02 BC	0.23 ± 0.05 BC	0.29 ± 0.02 AB	0.29 ± 0.03 AB	0.32 ± 0.09 AB	0.38 ± 0.01 A	0.34 ± 0.03 A
Quercetin 3-*O*-rhamnoarabinoside 7-*O*-rhamnoside	0.0014 ± 0 AB	0.0014 ± 0 AB	0.0013 ± 0.00 B	0.0014 ± 0 AB	0.0015 ± 0 AB	0.0015 ± 0 AB	0.0015 ± 0AB	0.0016 ± 0 A	0.0016 ± 0 A
Kaempferol 3-*O*-rhamnoarabinoside 7-*O*-rhamnoside	1.27 ± 0.2 E	1.59 ± 0.02 DE	1.56 ± 0.05 DE	1.7 ± 0.1 D	1.95 ± 0.1 CD	2.20 ± 0.1 BC	2.64 ± 0.3 A	2.58 ± 0.5 A	2.37 ± 0.2 AB
Kaempferol 3-*O*-acetyl-rhamnogalactoside 7-*O*-rhamnoside (isomer 1)	0.19 ± 0.06 C	0.21 ± 0.04 BC	0.22 ± 0.03 BC	0.25 ± 0.03 ABC	0.24 ± 0.04 BC	0.22 ± 0.02 BC	0.32 ± 0.08 AB	0.36 ± 0.03 A	0.28 ± 0.04 ABC
Kaempferol 3-*O*-acetyl-rhamnogalactoside 7-*O*-rhamnoside (isomer 2)	0.68 ± 0.1 E	0.76 ± 0.02 DE	0.64 ± 0.00 E	0.98 ± 0.1 CD	0.97 ± 0.1 CD	1.04 ± 0.1 BC	1.33 ± 0.1 A	1.28 ± 0.04 AB	1.08 ± 0.03 ABC
Kaempferol 3-*O*-rhamnoglucoside	0.024 ± 0.00 F	0.029 ± 0.00 EF	0.037 ± 0.00 DE	0.048 ± 0.00 BC	0.054 ± 0.00AB	0.043 ± 0.00 CD	0.053 ± 0.00 AB	0.057 ± 0.00 A	0.043 ± 0.01 CD

Different letters indicate significant differences within rows based on the Tukey HSD test, with *p* ≤ 0.05

In this study, the contrasting levels of sulfur and selenium differentially affected the flavonol glycosylated accumulation during faba bean–*Rhizobium* symbiosis.

### The impact of sulfur nutrition on flavonol glycoside accumulation

The findings of this study revealed that sufficient or higher sulfur treatment increased the concentration of kaempferol 3-*O*-acetyl-rhamnogalactoside 7-*O*-rhamnoside (isomer 1) by 31% and 49%, respectively (Table 3). Meanwhile, kaempferol 3-*O*-rhamnoglucoside 7-*O*-rhamnoside (isomer 1) increased with higher sulfur levels (by 50%) and no selenium application. Furthermore, kaempferol 3-*O*-rhamnoglucoside level increased (50%) with adequate sulfur level. Some studies have examined the impact of sulfur application on flavonoid accumulation in various food crops; however, the effect of sulfur application on flavonoid accumulation remains unclear. Most studies have investigated the effects of sulfur treatment on S-containing metabolites, such as glucosinolates and their hydrolysis products, as well as organosulfur compounds found in garlic. Accordingly, it has been found that sulfur supplementation enhanced glucosinolates in Brassica vegetables (Abdalla et al. 2020; Aghajanzadeh et al. 2014). Hence, flavonoids are biosynthesized via the phenylpropanoid pathway; there is little understanding regarding the impact of sulfur application on the synthesis of flavonoids in different food crops, with conflicting results among the few published data. For instance, quercetin and its glycoside derivatives increased during sulfur-deficient conditions in lettuce plants (Abdalla et al. 2021), which is not shown in the current study. This might be attributed to the different experimental conditions, where the faba bean in this study is grown under N_2_fixation. However, quercetin glycoside concentrations remained unaltered under sulfur limitation. Previous studies have also demonstrated that sulfur supply increased the total phenolic content and antioxidant potential in the leaves of some food crops (De Pascale et al. 2007; Vallejo et al. 2003). Here the data confirm this for specific kaempferol glycosides of faba bean such as kaempferol 3-*O*-acetyl-rhamnogalactoside 7-*O*-rhamnoside (isomer 1), kaempferol 3-*O*-rhamnoglucoside 7-*O*-rhamnoside (isomer 1), and kaempferol 3-*O*-rhamnoglucoside. (Nikiforova et al. 2005)indicated that flavonoids and their subclasses displayed higher concentrations in Arabidopsis under sulfur-deficient conditions (Nikiforova et al. 2005). However, Kim et al. (2021) showed that flavonoid accumulation in bell pepper leaves demonstrated relatively differential responses under sulfur-deficient conditions. The authors stated that luteolin 7-*O*-(2-*O*-apiosyl)glucoside, together with two unknown compounds (related to apigenin-derivative), diminished by S starvation, however, luteolin 7-*O*-(2-*O*-apiosyl-6-*O*-malonyl) glucoside, along with another two unknown compounds (related to apigenin-derivatives) enhanced under sulfur limitation. Regarding the impact of sulfur on flavonoid levels under legume–rhizobium symbiotic conditions, previous research stated that flavonoids increased slightly without significant differences following elevated sulfur treatment in the model legume *Lotus japonicus *(Siegl et al. 2024). The authors indicated that shoot metabolites remained in a nonstress response under sulfur deficiency, which can be attributed to the long-distance transport of sulfur from the aboveground plant to root nodules, playing a key role in augmenting the maintenance of symbiotic N_2_ fixation in response to demand-driven sulfur distribution. Hence, sulfur and N are necessary for protein synthesis, including enzymes, which are required in the biochemical processes.

Notably, kaempferol 3-*O*-rhamnogalactoside 7-*O*-rhamnoside and quercetin 3-*O*-rhamnoarabinoside 7-*O*-rhamnoside remained unaffected by different selenium and sulfur levels, which can be attributed to the importance of flavonoids under stress conditions, where the plant can biosynthesize them under sulfur deficiency to acclimate to these inconvenient conditions. For instance, they might act as antioxidants. Consequently, their levels did not even decrease under sulfur deficiency levels (S0). A previous study mentioned that plants have different mechanisms for mitigating the negative effects of various abiotic stresses (Haak et al. 2017), but the mechanisms themselves are not yet completely understood. These mechanisms include physiological interferences, such as the regulation of stomatal movements by the abscisic acid signaling mechanism and ionic and osmotic homeostasis signaling pathways, which lead to plant acclimation under drought and salt stress (Kuromori et al. 2022). Moreover, the antioxidant defense system diminished heat-induced oxidative damage (Mittler et al. 2022). Flavonoids constitute a metabolic pathway for plant acclimation under stress conditions by different modes of action, including scavenging free radicals, chelating metal ions, and triggering plants’ natural antioxidant enzymes (Procházková et al. 2011; Shah and Smith 2020).

### The impact of selenium considering sulfur availability on flavonol glycosides accumulation

The findings revealed that kaempferol 3-*O*-rhamnoarabinoside 7-*O*-rhamnoside showed up in much higher quantities in response to adequate or higher sulfur levels with lower or higher selenium levels (by 53% and 51%, respectively). Additionally, kaempferol 3-*O*-acetyl-rhamnogalactoside 7-*O*-rhamnoside (isomer 2) increased with higher sulfur and lower selenium levels (by 47%). Moreover, lower selenium and adequate sulfur levels or higher selenium and sulfur treatments enhanced the concentrations of quercetin 3-*O*-rhamnoglucoside 7-*O*-rhamnoside (by 13.3% and 18.8%), kaempferol 3-*O*-rhamnoglucoside 7-*O*-rhamnoside 4′-*O*-rhamnoside (isomer 2) (by 48% and 60%), and kaempferol 3-*O*-rhamnoglucoside 7-*O*-rhamnoside (isomer 2) (by 38% and 52%). Hence, selenium can be assumed to be beneficial for the induction of flavonoid biosynthesis, rendering selenium biofortification a sustainable and effective approach that not only increases plant defense against environmental stresses and the nutritional quality of crops but also boosts human health (Banuelos et al. 2023; Wang et al. 2022). Selenium has antioxidant properties that protect cells against oxidative damage, improving the immune system, cognitive function, and thyroid health, as well as reducing cancer risk and cardiovascular problems (Bai et al. 2025).

**Fig. 3:**
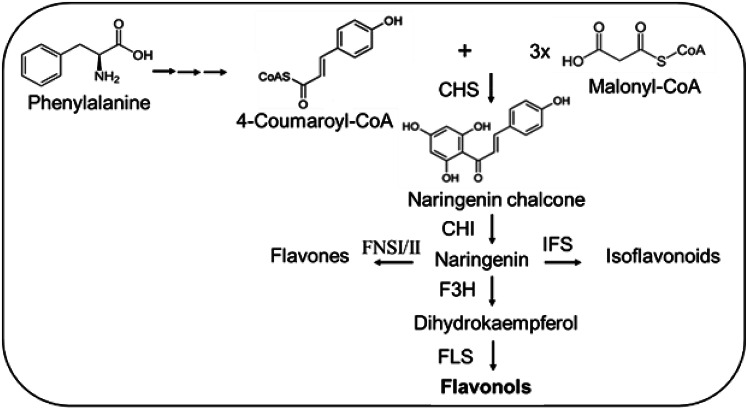
The biosynthetic pathway of flavonols is derived from the phenylpropanoid pathway. Enzymes: *CHS* chalcone synthase, *CHI* chalcone isomerase, *IFS* isoflavone synthase, *FNSI*/*II* flavone synthase 1 or 2, *F*3*H* flavanone 3-hydroxylase, *FLS* flavonol synthase

Several studies agree with the current findings, as they have proven that selenium application can affect the metabolomic characteristics of legumes, especially the biosynthesis of flavonoids. For instance, (Zhao et al. 2024) studied the impact of selenium by treating seeds with 20 µM sodium selenite on regulating flavonoid biosynthesis in sprouts of different leguminous plant mung bean (*Vigna radiata*) genotypes. The authors indicated that selenium application promoted flavonoid metabolism and antioxidant capacity during germination, which has been attributed to the induction of the biosynthesis of the major mung bean flavonoids (e.g. vitexin, and isovitexin), which were more common in some genotypes. The findings of the present investigation suggested that selenium might stimulate CHS and CHI (Fig. 3), to produce naringenin, which is known as the common precursor for *C*-glycosylated flavones and other flavonoids, which agreed with (Zhao et al. 2024). (Wang et al. 2022) studied the impact of varied selenium applications (e.g. four selenium levels named 0 (CK), 15 (Y1), 30 (Y2), and 45 (Y3) g ha^−1^) on nutritional quality and metabolomic changes in two mung beans varieties (P1 and P2) using an LC-MS/MS-based targeted metabolomics approach. The authors indicated that the selenium foliar application of 30 g ha^−1^ increased the total phenol and flavonoid content in mung bean varieties. The authors detected 92 compounds—including amino acids and their derivatives, phenolic acids, flavonoids, and lipids—in response to different selenium levels (0 and 30 g ha^−1^) in the P1 variety; among them, 53 metabolites increased, and 39 decreased. In the P2 variety (between selenium levels of 0 and 30 g ha^−1^), 72 metabolites were deduced—mainly flavonoids and phenolic acids—among which 30 increased, and 42 decreased (Wang et al. 2022). Concerning flavonoid accumulation in the P1 variety, the authors identified the patterns of the expression of tamarixetin 3-*O*-glucoside and isovitexin 8-oxyloside, which increased in a constant selenium treatment. Consequently, in the P2 variety, isoluteolin 6,8-di-C-glucoside accumulation was concurrently enhanced following selenium application (Wang et al. 2022).

These findings agree with those of the current study, which indicates the beneficial impact of selenium on flavonoid biosynthesis in legumes. However, to the best of our knowledge, this is the first study to show the impact of selenium and sulfur enrichment on flavonoid accumulation during faba bean–*Rhizobium* symbiosis.

## Conclusion

Flavonol glycosides are bioactive compounds that constitute an important subclass of flavonoids. Moreover, due to their various beneficial effects, including considerable antioxidative capacities, flavonol glycosides can be of great interest in the food industry. The current study offers new insights into enhancing flavonol glycosides through selenium and sulfur enrichment under faba bean–*Rhizobium* symbiosis, thereby paving the way for further research on the nutritional significance of faba beans. Furthermore, this approach could enhance the health benefits of Se-enriched legumes, making them a significant source of bioactive compounds suitable for use as plant-based meat alternatives in human nutrition. Further studies should, therefore, be considered to elucidate the molecular mechanism underlying the potential role of selenium in enhancing flavonoid glycosides under faba bean–*Rhizobium* symbiosis. To discover the regulatory mechanism a comprehensive metabolomic, together with transcriptomic studies, remains necessary to increase the in-depth understanding of the relationship between the key precursors of primary metabolites and the phenylpropanoid pathway, which is related to the biosynthesis of phenolic acids, including all classes of flavonoids, under the enrichment of selenium and sulfur.

## Data Availability

No datasets were generated or analysed during the current study.
